# Low Temperature Epitaxial LiMn_2_O_4_ Cathodes Enabled by NiCo_2_O_4_ Current Collector for High-Performance Microbatteries

**DOI:** 10.1021/acsenergylett.3c01094

**Published:** 2023-07-18

**Authors:** Adam J. Lovett, Venkateswarlu Daramalla, Farheen N. Sayed, Debasis Nayak, Muireann de h-Óra, Clare P. Grey, Siân E. Dutton, Judith L. MacManus-Driscoll

**Affiliations:** † Department of Materials Science and Metallurgy, University of Cambridge, 27 Charles Babbage Road, Cambridge CB3 0FS, United Kingdom; ‡ Cavendish Laboratory, University of Cambridge, JJ Thompson Avenue, Cambridge CB3 0HE, United Kingdom; § The Faraday Institution, Quad One, Harwell Campus, Didcot OX11 0RA, United Kingdom; ∥ Yusef Hamied Department of Chemistry, Lensfield Rd., Cambridge CB2 1EW, United Kingdom

## Abstract

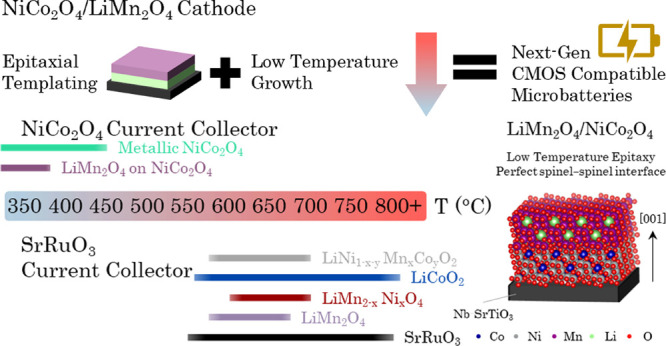

Epitaxial cathodes in lithium-ion microbatteries are ideal model systems to understand mass and charge transfer across interfaces, plus interphase degradation processes during cycling. Importantly, if grown at <450 °C, they also offer potential for complementary metal–oxide–semiconductor (CMOS) compatible microbatteries for the Internet of Things, flexible electronics, and MedTech devices. Currently, prominent epitaxial cathodes are grown at high temperatures (>600 °C), which imposes both manufacturing and scale-up challenges. Herein, we report structural and electrochemical studies of epitaxial LiMn_2_O_4_ (LMO) thin films grown on a new current collector material, NiCo_2_O_4_ (NCO). We achieve this at the low temperature of 360 °C, ∼200 °C lower than existing current collectors SrRuO_3_ and LaNiO_3_. Our films achieve a discharge capacity of >100 mAh g^–1^ for ∼6000 cycles with distinct LMO redox signatures, demonstrating long-term electrochemical stability of our NCO current collector. Hence, we show a route toward high-performance microbatteries for a range of miniaturized electronic devices.

Current collectors are a vital component of lithium-ion batteries (LIB), enabling the improvement of electrical conductivity and reduction in contact resistance, thus enhancing the performance of the LIB.^1^ For epitaxial thin film cathodes, typically grown by pulsed laser deposition (PLD) on electronically conducting Nb-doped SrTiO_3_ (Nb-STO) single-crystal substrates, current-collecting buffer layers are a necessary component, without which distinct cathode redox behavior may not be observed.^2,3^ For this purpose, the perovskites SrRuO_3_ (*a* = 3.93 Å) and LaNiO_3_ (pseudocubic *a* = 3.83 Å) have seen wide use,^2,4−10^ due to exhibiting metallic resistivity (<1 mΩ cm at 25 °C) and a small lattice mismatch with Nb-STO (∼1%).^11−14^ Low resistivity (ρ < 1 mΩ cm at operational temperature), bandgap alignment, and epitaxial growth on the substrate of choice form the key criteria for a current-collecting buffer layer in an epitaxial thin film battery. However, not every electronic conducting oxide can be used; a recent study attempted to use a perovskite La_0.5_Sr_0.5_CoO_3_ (LSCO) current collector in a LiMn_2_O_4_/LSCO/Nb-STO film stack. No redox peaks were observed beyond the first cycle, attributed to irreversible lattice oxygen loss in the LSCO layer during the first charging cycle, inducing a 10× reduction in electronic conductivity.^15^

The selection of the right electronic conducting phase is important, as the heterojunction substrate/current-collector/cathode interfaces can be highly resistive and rectifying, in turn inhibiting the electrochemical performance of the battery.^3,11,16−18^ The current collector must also withstand the cycling conditions.^15^ Further, the current-collecting buffer layer influences the thermodynamically favorable phase, orientation, and epitaxial nature of the cathodic material grown upon it. Thus, if the cathode film is astructural to the current collector, it will be more defective and less stable, particularly at the interfacial region. On the other hand, if isostructural and closely lattice matched current collector/cathode combinations are used, the cathode and its interface with the current collector/substrate will be much more perfect.^19^ Equally important, the cathode could be grown at lower temperatures, with the phase stabilization being enabled by the epitaxial templating. This is very important for LiMn_2_O_4_ (LMO) and LiMn_2–*x*_Ni_*x*_O_4_ (LMNO) cathodes (typically grown at ≥600 °C^3,4,6,8,20^) as high growth temperatures promote a loss of volatile lithium, inducing the formation of lithium-deficient phases and off-stoichiometric lithium-content films, both which can severely impact the electrochemical performance of the resulting cathode.^20,21^ Using perovskite SrRuO_3_ (SRO) current collectors, as showcased in Figure 1a, the substrate temperatures required to achieve epitaxial cathodes are high (>600 °C, Figure 1). Hence, identification of a current-collecting buffer layer that could promote low temperature (<400 °C) epitaxial growth of prominent cathodes, such as the spinels LiMn_2_O_4_ (LMO) and LiMn_2–*x*_Ni_*x*_O_4_ (LMNO), would be highly desirable.

**Figure 1:**
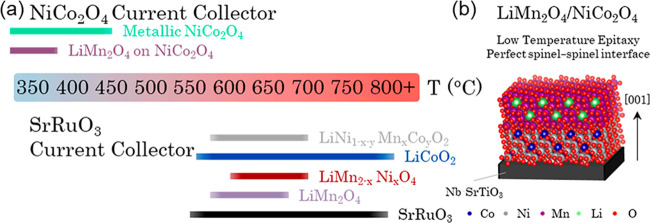
(a) Schematic illustrating the PLD thin film growth temperatures of prominent cathodes on SrRuO_3_/Nb-SrTiO_3_ substrates, currently restricted to temperatures >600 °C. Utilizing NiCo_2_O_4_ enables epitaxial growth of LiMn_2_O_4_ at 360 °C. (b) Advantages of using a NiCo_2_O_4_ current collector that is isostructural to LiMn_2_O_4_. The LiMn_2_O_4_/NiCo_2_O_4_ interface will be less defective and thus more stable, enabling good adhesion and improved electro-chemo-mechanical stability.

We propose that NiCo_2_O_4_ (NCO) could be used to replace SRO and LaNiO_3_ for stabilization of isostructural LMO and LMNO at low growth temperatures, ideally at the complementary metal–oxide–semiconductor (CMOS) compatible temperature of <450 °C. NCO has seen use in a range of fields including supercapacitors,^22−24^ battery anodes,^25,26^ solid oxide fuel cell cathodes,^27^ and transparent conducting oxides in water splitting devices.^28^ NCO belongs to the spinel family of materials but is known to display cation disorder, where the Ni and Co cations can readily exchange between the tetrahedral (Td) and octahedral (Oh) sites.^29,30^ Hence, the NCO formula is more generally written as (Co_λ_Ni_1−λ_)_Td_[Co_2−λ_Ni_λ_]_Oh_O_4_, where the fraction (λ: 0 ≤ λ ≤ 1) of Ni in the octahedral site is referred to as the degree of inversion (DOI).^29^ When λ = 1, NCO adopts an inverse spinel configuration, whereas when λ = 0 it adopts a normal spinel. All other configurations (0 < λ < 1) are intermediate structures and can be regarded as a combination of both the inverse and normal spinel structures with different ratios.^31^ The DOI influences both the lattice constant^32,33^ and electronic properties^34,35^ of the resultant NCO; PLD thin films grown above 500 °C display insulating behavior, whereas films grown below 450 °C are metallic (ρ < 1 mΩ cm at 300 K), exhibiting a metal–insulator transition around 50 K.^34,36^ The DOI can be altered by annealing films at elevated temperatures post-deposition.^29^ Crucially, the fact that epitaxial metallic conducting NCO is achievable at low temperatures (<400 °C) makes it an interesting candidate for a current-collecting buffer layer in lithium-ion microbatteries.

Herein, we report for the first time structural and electrochemical studies of low temperature epitaxial LMO films grown on the novel current collector NCO. First, we demonstrate that epitaxial growth of LMO can be achieved on NCO at 360 °C, ∼200 °C lower than previous LMO/SRO/Nb-STO systems.^3,4^ Then, we showcase clear reversible LMO redox behavior, demonstrating longevity with a high discharge capacity (>100 mAh g^–1^) for ∼6000 cycles. These results affirm that NCO is a promising alternative current-collecting buffer layer for low temperature epitaxial batteries within the thermal stability range of CMOS technologies.

We start by first characterizing the orientation and lattice parameter of a planar NCO film grown on Nb-STO (001). High resolution X-ray diffraction (XRD) symmetric 2θ–ω scans were undertaken (Figure 2a). The peaks are indexed to a (00l) oriented cubic NCO phase with an out-of-plane lattice parameter of *a* = 8.20(1) Å. From φ scans (Supplementary Figure S1b), we confirm that NCO grows epitaxially with the relationship: [011]NCO//[011]Nb-STO. Also, from reciprocal space maps (RSM) of the STO(113)/NCO(226) reflections (Supplementary Figure S1c), we determine the in-plane lattice parameter to be *a* = 8.198(5) Å, which are closely matched to the out-of-plane lattice parameter and confirm the cubic nature of the film.

**Figure 2:**
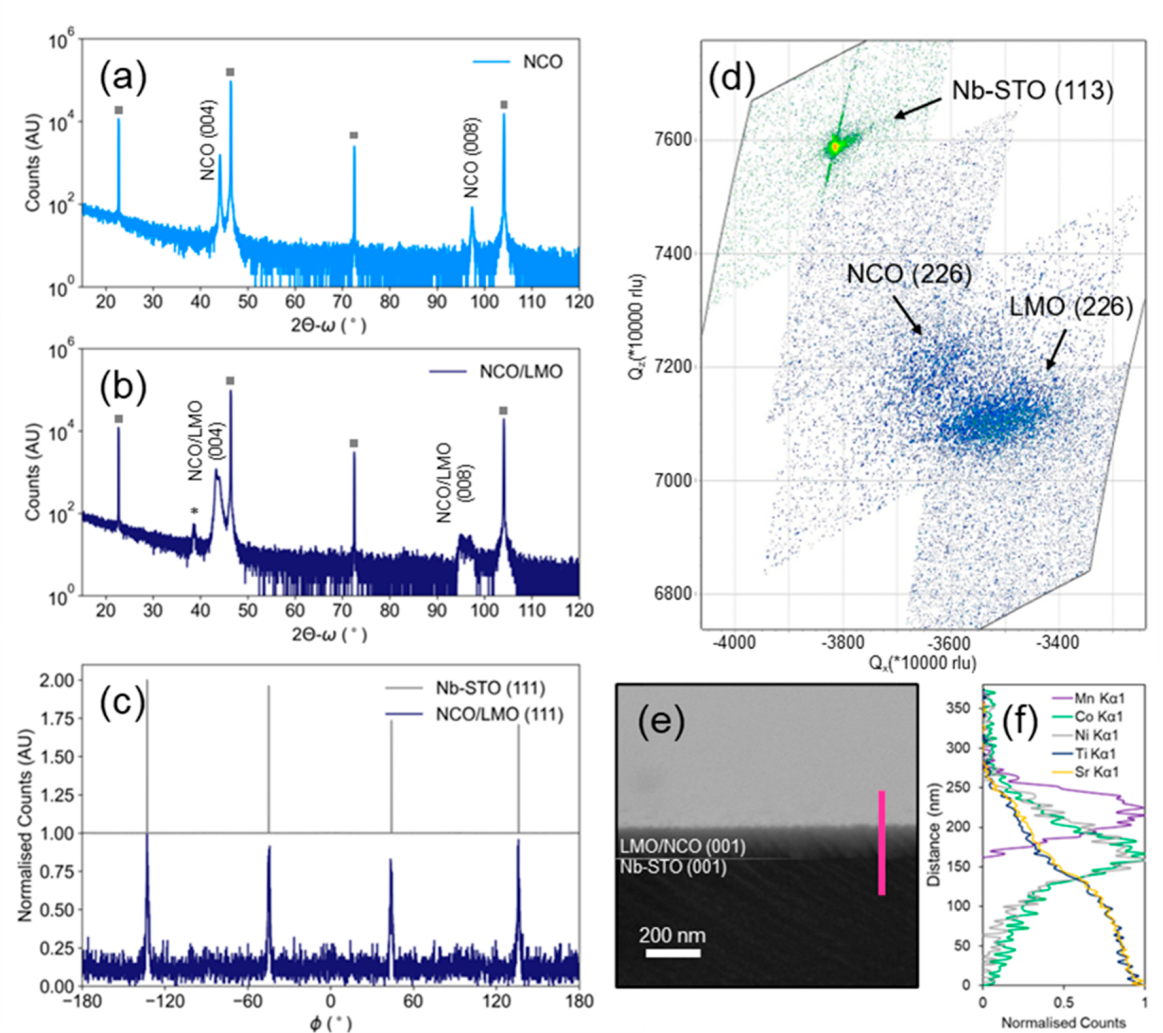
Structural characterization of LMO/NCO films grown on Nb-STO (001) (a,b) 2θ–ω scan of (a) planar NCO (001) and (b) LMO/NCO (001) films grown on Nb-STO (001). All films are epitaxial with the out-of-plane relationship: LMO/NCO(00l)//Nb-STO(00l). Reflections marked by gray squares correspond to Nb-STO (00l) substrate reflections. The additional reflection marked by an * in (b) is ascribed to a small impurity (<5%) of tetragonal Li_2_Mn_2_O_4_. (c) φ-scans of LMO/NCO film on Nb-STO (001), confirming the in-plane relationship: LMO/NCO[111]//Nb-STO[111]. Due to close overlap of NCO and LMO reflections, they cannot be deconvoluted. (d) RSM of LMO/NCO around the Nb-STO (113) reflection. Two broad reflections are observed, assigned to LMO/NCO (226). (e) Cross-sectional SEM of LMO/NCO film with (f) the corresponding EDX line scan taken from the pink line in (e).

We note that our observed lattice parameters are larger than bulk NCO (*a* = 8.12 Å^34^), which is similar to previously reported PLD grown NCO films on MgAl_2_O_4_ substrates (in-plane *a* = 8.08 Å, out-of-plane *c* = 8.17–8.20 Å^34^). This is attributed to cation disorder of Ni/Co inducing variations in the as-grown tetrahedral/octahedral occupancies, in turn altering the lattice parameter.^32−34^ From scanning electron microscopy (SEM) imaging, the film thickness is determined to be 80 nm (Supplementary Figure S2), corresponding to a growth rate of 240 nm h^–1^.

Next, we confirm the epitaxial nature of an LMO film grown at 360 °C within an LMO/NCO/Nb-STO film stack. In symmetric 2θ–ω XRD scans (Figure 2b), two sets of overlapping reflections (Δ2θ < 0.3°) are observed, assigned to the (00l) reflections of NCO and LMO. Their out-of-plane lattice parameters are determined to be *a*_NCO_ = 8.23(1) Å and *a*_LMO_ = 8.34(1) Å, respectively. φ scans (Figure 2c) confirm the epitaxial nature of both NCO and LMO (which cannot be separated due to the near-perfect lattice matching epitaxy) with the relationship: LMO/NCO[111]//Nb-STO[111]. From RSMs collected around the (113) Nb-STO reflection (Figure 2d), the in-plane lattice parameters are determined to be *a*_NCO_ = 8.225 Å and *a*_LMO_ = 8.360(5) Å, closely matched to the out-of-plane parameters and confirming that both phases are relaxed and exhibit cubic structures. The film microstructure, studied via SEM (Figure 2e), consists of a dense film stack totaling 120 nm (80 nm NCO, 40 nm LMO). Clear phase separation of the NCO and LMO phases is confirmed via separation of the Mn and Ni/Co peaks in an SEM-EDX line scan (Figure 2f), taken from the pink line in Figure 2e.

We note that here our LMO film grown on Nb-STO (001) at 360 °C exhibits larger lattice parameters (*a*_LMO_ = 8.36 Å) than typically observed for LMO PLD thin films grown on SRO (8.15 Å < *a*_LMO_ < 8.25 Å^3−5^). Also, there is an additional reflection (marked by an asterisk in Figure 2b) at ∼38.5°, *c* = 9.35(1) Å which is assigned to a small impurity (<5%) corresponding to the lithium-rich tetragonal Li_2_Mn_2_O_4_ phase (*a* = 5.66 Å, *c* = 9.22–9.33 Å^37,38^). Both observations are a direct consequence of the formation of an over-lithiated LMO film which arises because our PLD target, which contains a nominal composition of Li_1.2_Mn_2_O_4_ (20% molar excess of lithium), was optimized to account for lithium loss at growth temperatures between 500–600 °C.^39^ We stress that the presence of over-lithiated LMO is a positive observation, as it demonstrates that lower temperature PLD leads to a reduction in lithium loss, a key advantage of growing epitaxial cathodes at lower temperatures.

We now turn our attention to the electrochemical performance of our LMO/NCO films grown at 360 °C. Galvanostatic charge–discharge cycling and rate performance (Figure 3 and Figure 4) were tested sequentially with the cycling conditions outlined in Supplementary Table S1. The corresponding galvanostatic charge–discharge curves (Figure 3a) and differential capacity (dQ/dV) profiles (Figure 3b) display the characteristic LMO redox processes. The first charge–discharge curve (black lines, Figure 3a) exhibits a higher charging capacity, with some irreversible processes occurring totaling a capacity of ∼43 mAh g^–1^. All subsequent cycles display the distinct reversible electrochemical redox process of LMO: two peaks between ∼4.0–4.2 V vs. Li/Li^+^ corresponding to the reversible two stage lithiation of LiMn_2_O_4_ via the Mn^3+^/Mn^4+^ redox couple.^40^ A specific capacity of >100 mAh g^–1^ (>1.65 μAh cm^–2^) is achieved for ∼6000 cycles (Figure 4a), irrespective of the current density tested (4–40 μA cm^–2^, ∼2.2–23 C). Under the first cycling condition (40 μA cm^–2^), the LMO/NCO film stack displays an initial discharge capacity of 103 mAh g^–1^, falling to 93 mAh g^–1^ after ∼300 cycles before gradually rising to 111 mAh g^–1^ after 2000 cycles. Upon switching to 20 μA cm^–2^ (cycles 2000–4000, Figure 4a), our film displays an initial discharge capacity of 124 mAh g^–1^, gradually fading to 108 mAh g^–1^ (cycle 4000). The cell was then allowed to equilibrate at open current voltage for 12 h before being cycled at 4 μA cm^–2^ (cycle 4000–6000) with a stable discharge capacity between 95–110 mAh g^–1^. This cycle performance (Figure 4a) demonstrates the long-term cycling stability of our NCO current collector. We also note that it can be assumed that our calculated gravimetric capacities are underestimated. This is because our calculation incorporates conservative estimates for the film density (4.3 g cm^–3^, bulk density of LMO) and thickness, the surface roughness complicating assessment^41^ (see Experimental Section in the Supporting Information). Compared with SRO, three (001) oriented planar LMO(110 nm)/SRO/Nb-STO films reported in the literature cycled at 5 μA (20 μA cm^–2^) exhibited initial capacities of 120–130 mAh g^–1^, gradually fading to ∼90 mAh g^–1^ after 1000 cycles.^4^ Hence, our LMO/NCO cathode displays an improvement in cycle performance (up to a 20% increase in discharge capacity) under comparable cycling conditions after 3 times the number of cycles.^4^

**Figure 3:**
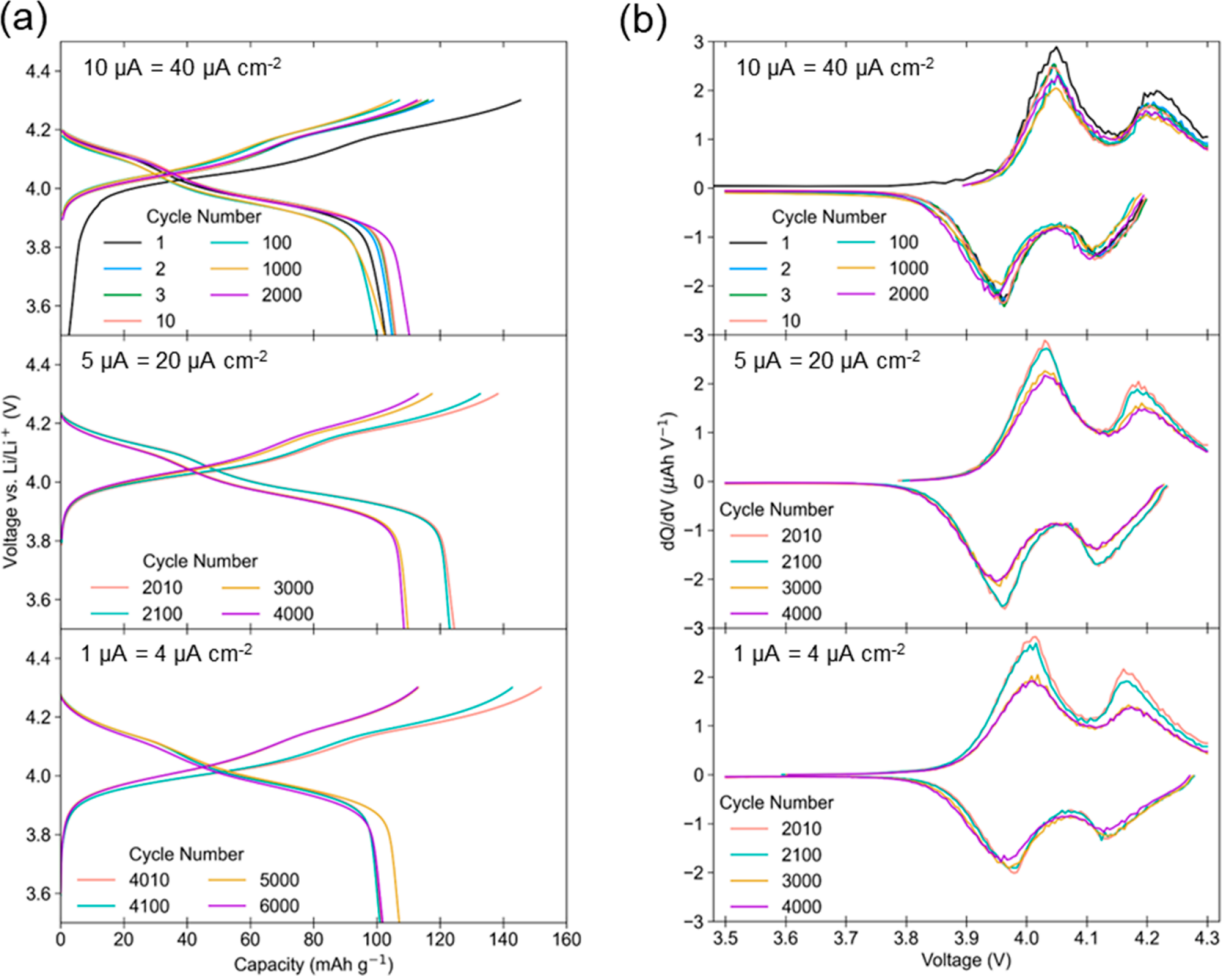
Accompanying (a) charge–discharge curves and (b) differential capacity (dQ/dV) profiles to the cycle performance presented in Figure 4a. All charge–discharge curves and differential capacity profiles display the characteristic redox behavior of LMO.

**Figure 4:**
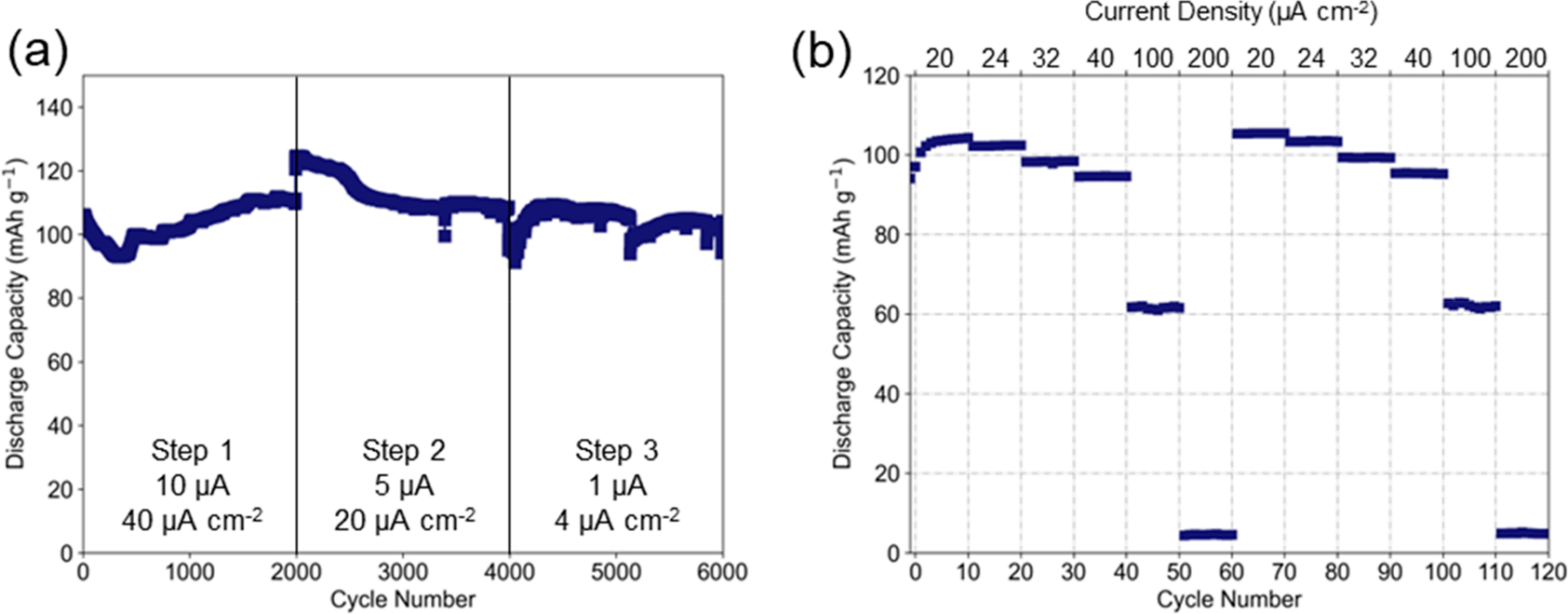
Electrochemical performance of LMO/NCO films grown on Nb-STO. (a) Discharge capacity obtained from galvanostatic cycling under three current regimes: 40, 20, and 4 μA cm^–2^, cycled between 3.5 and 4.3 V vs Li/Li^+^. Each galvanostatic cycling test was performed sequentially. (b) Rate dependent electrochemical performance conducted post galvanostatic charge–discharge cycling. Two initial pre-cycles at 20 μA cm^–2^ were performed followed by two rounds of rate testing (cycles 1–60 and 61–120) between 20 and 200 μA cm^–2^. The recovery of the discharge capacity at the beginning of round 2 affirms the cycling stability of the NCO current collector.

The rate performance (Figure 4b) was also assessed after 6000 cycles. The film shows good cycle stability, as demonstrated by the recovery of discharge capacity after exposure to high current densities. A capacity retention of >90% is achieved for current densities up to 40 μA cm^–2^ (∼23 C) with a discharge capacity of >95 mAh g^–1^. Again, it should be noted that this rate performance test was conducted after 6000 cycles, with the sample displaying signs of capacity fade; hence, a pristine LMO/NCO sample would be expected to exhibit higher discharge capacities. But despite this, the recovery of the discharge capacity exemplifies the long-term stability of our low temperature epitaxial LMO/NCO cathode/current-collecting buffer layer film stack, even after significant testing under high-rate conditions.

In conclusion, we demonstrate, for the first time, the growth of an epitaxial LMO cathode utilizing a novel NCO current-collecting buffer layer on Nb-STO. By using NCO, we decrease the epitaxial growth temperature range of LMO from ∼600 °C (for SRO) to 360 °C. First, we confirm the epitaxial nature of both NCO and LMO grown at 360 °C. Thereafter, we demonstrate the electrochemical performance of our LMO/NCO/Nb-STO (001) system, achieving a discharge capacity of >100 mAh g^–1^ for ∼6000 cycles, a modest improvement over LMO/SRO/Nb-STO (001) films grown at higher temperatures. By facilitating low temperature growth, our work marks an important advance toward the implementation of epitaxial cathodes in next-generation microbatteries that could be deployed in flexible electronics, the Internet of Things, and MedTech devices.

## Supporting Information Available

Experimental section and additional characterization of NiCo_2_O_4_ thin films on Nb-SrTiO_3_ (XRD: 2θ–ω, φ-scan and RSM, and cross-sectional SEM) (PDF)
