# Supplementary material for: Low Temperature Epitaxial LiMn2O4 Cathodes Enabled by NiCo2O4 Current Collector for High-Performance Microbatteries
Source: ACS Energy Lett. 2023 Jul 18;8(8):3437–42. doi: 10.1021/acsenergylett.3c01094 (PMC10425970; doi:10.1021/acsenergylett.3c01094)
Supplement: Supplementary file 1 — nz3c01094_si_001.pdf [file nz3c01094_si_001.pdf]

# Supplementary Information: Low Temperature Epitaxial $LiMn_2O_4$ Cathodes Enabled by $NiCo_2O_4$ Current Collector for High-Performance Micro-Batteries

Adam J. Lovett[1], Venkat Daramalla[2,3], Farheen N. Sayed [3,4], Debasis Nayak[1,3], Muireann de h-Óra[1], Clare P. Grey [3,4], Siân E. Dutton[2,3], Judith L. MacManus-Driscoll[1]

[1]Department of Materials Science and Metallurgy, University of Cambridge, 27 Charles Babbage Road, Cambridge CB3 0FS, United Kingdom.

[2]Cavendish Laboratory, University of Cambridge, JJ Thompson Avenue, Cambridge CB3 0HE, United Kingdom.

[3]The Faraday Institution, Quad One, Harwell Campus, Didcot, OX11 0RA, United Kingdom

[4]Yusef Hamied Department of Chemistry, Lensfield Rd, Cambridge CB2 1EW, United Kingdom.

## Experimental

### Target Preparation

**$NiCo_2O_4$**: Stoichiometric ratios of NiO and CoO were hand-ground in a pestle and mortar, then pelletised with a diameter of 25 mm and height of approximately 3 mm. Then, the target was sintered at 1000 °C for 8 hours. After, the target surface was sanded before being mounted on an aluminium disc backing plate using carbon tape.

**$LiMn_2O_4$**: LiMn2O4 (Sigma Aldrich) and $Li_2O$ with a Li:Mn ratio of 0.6 (20 % molar excess of Li) were mixed on a ball mill for 30 minutes then pelletised with a diameter of 13 mm and height of approximately 2 mm. Then, the target was sintered at 900 °C for 10 hours. After, the target was mounted on an aluminium disc backing plate using carbon tape.

### Pulsed Laser Deposition

Films were grown by pulsed laser deposition (PLD) from the sintered LMO/NCO targets using a KrF excimer laser with a wavelength of 248 nm. Before each deposition, the chamber was evacuated to at least $10^{-5}$ Pa before being filled with oxygen. Substrates were cleaned with acetone in an ultrasonic bath for 5 min and mounted onto a substrate carrier with the silver paste (pre-heated to set at 100 °C). NCO and LMO thin films were grown on (001) orientated SrTiO3/Nb-doped SrTiO3 (Nb-STO) (0.5 wt. % Nb) substrates (5 x 5 $mm^2$) purchased from CrysTec GmbH. The optimised deposition conditions are as follows:

NCO) $T_{sub}$ = 360 °C, F = 2.3 J $cm^{-2}$ , $pO_2$ = 6.6 Pa, v = 10 Hz, $Z_{height}$ = 57 mm, pre-ablation of 1000 shots at 5 Hz; LMO) $T_{sub}$ = 360 °C, F = 2.3 J $cm^{-2}$ , $pO_2$ = 13 Pa, v = 2 Hz, $Z_{height}$ = 45 mm, pre-ablation of 450 shots at 5 Hz. Films were annealed in $pO_2$ = 0.2 bar during cooling.

## Structural Characterisation

**X-Ray Diffraction:** Films were characterised with high resolution X-ray diffraction (XRD) performed on a Panalytical Empyrean vertical diffractometer using a Cu $K_\alpha$ X-ray radiation source with a wavelength of 1.5418 Å.

**Electron Microscopy:** Film thicknesses were characterised by scanning electron microscopy (SEM) and energy dispersive X-ray spectroscopy (EDS) on a TESCAN MIRA3 FEG-SEM. SEM samples were prepared by sputtering a 10 nm thick Au layer onto the surface to be imaged, mounted onto an SEM stub with carbon tape, and then surface-grounded with copper tape. Cross-sectional SEM samples were prepared by scoring the backside of samples with a diamond scribe and then snapping them, before mounting.

## Electrochemical Characterisation

For all electrochemical characterisation, LMO/NCO films grown on Nb-STO (001) were transferred to an argon atmosphere glovebox (< 0.5 ppm $H_2O$ and $O_2$) and placed on a hotplate for 10 min at 125 °C to remove any water content. Subsequently, cells were assembled in EL-Cell PAT Cells and combined with stainless steel plungers, a Whatman glass fibre separator, 60 µL electrolyte (1 M $LiPF_6$ in 1:1 ethylene carbonate/diethyl carbonate (EC/DEC)), and a pre-polished lithium metal disc anode (Pi-kem). Electrochemical measurements were conducted on an Arbin LBT20084 potentiostat in a two-electrode setup. Galvanostatic charge-discharge cycling was conducted between 3.5 - 4.3 V *vs.* Li/$Li^+$ on a sample with the cycling parameters outlined in Table S1. Then, a rate capability test was performed sequentially at currents between 5-125 µA (20-200 µA $cm^{-2}$) and operated between 3.5 - 4.3 V *vs.* Li/$Li^+$. The gravimetric capacity, $C_g$, is given by the following equation:

$$C_g(mAh\, g^{-1}) = \frac{Measured\ Capacity\ (mAh)}{\rho_{LMO}\ (g\ cm^{-3})\ \times\ film\ thickness\ (cm)\ \times\ film\ area\ (cm^2)}$$

where $\rho_{LMO}$ is the theoretical density of LMO (4.3 g $cm^{-3}$) and the film area is 0.25 $cm^2$ (the substrate size).

Table S1: Galvanostatic charge-discharge cycling parameters of LMO/NCO/Nb-STO system cycled between 3.5–4.3 V *vs.* Li/$Li^+$.

| Step Number (#) | Current (µA) | Current Density (µA $cm^{-2}$) | C Rate ($h^{-1}$) |
|---|---|---|---|
| 1 | 10 | 40 | 23 |
| 2 | 5 | 20 | 10 |
| 3 | 1 | 4 | 2.2 |

## Supplementary Figures

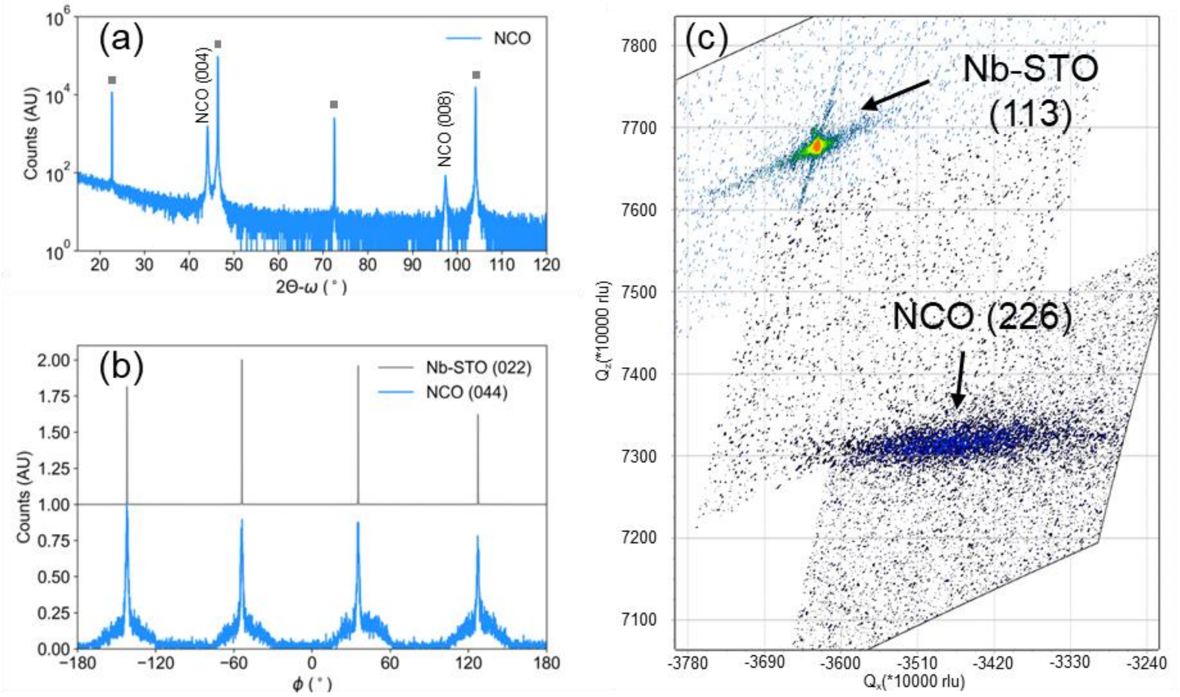


Figure S1: XRD of a planar NCO film grown on Nb-STO (001) (a) 2θ-ω scan of NCO (001) film grown on Nb-STO (001). Reflections marked by grey squares correspond to Nb-STO (00l) substrate reflections. (b) φ scan confirming epitaxial nature of NCO planar films with the relationship: [011]NCO//[011]Nb-STO. (c) Reciprocal space map of the NCO(226) reflection around Nb-STO (113) reflection.

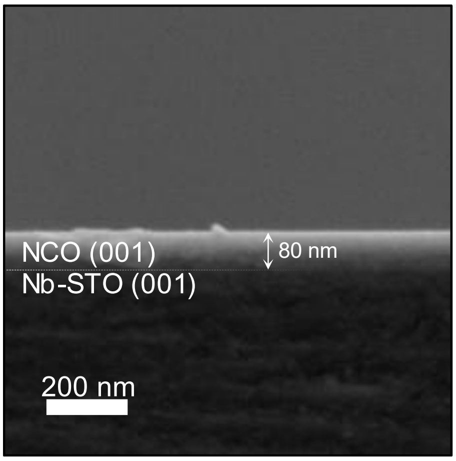


Figure S2: Cross-sectional SEM of a planar NCO (001) film grown on a Nb-STO (001) substrate. The film has a thickness of 80 nm.
